# Clinical-anatomic mapping of the tarsal tunnel with regard to Baxter’s neuropathy in recalcitrant heel pain syndrome: part I

**DOI:** 10.1007/s00276-018-2124-z

**Published:** 2018-10-27

**Authors:** Simone Moroni, Marit Zwierzina, Vasco Starke, Bernhard Moriggl, Ferruccio Montesi, Marko Konschake

**Affiliations:** 1 Minimally invasive Foot and Ankle Surgery, Faculty of Physical Therapy and Podiatry, Catholic University Saint Vincent Martyr, Valencia, Spain; 2 Faculty of Health Sciences at Manresa, Universitat de Vic-Universitat Central de Catalunya (Uvic-Ucc), Barcelona, Spain; 3 Department of Plastic, Reconstructive and Aesthetic Surgery, Center of Operative Medicine, Medical University of Innsbruck, Innsbruck, Austria; 4 Department of Anatomy, Histology and Embryology, Division of Clinical and Functional Anatomy, Medical University of Innsbruck, Müllerstr. 59, 6020 Innsbruck, Austria; 5 Faculty of Health Sciences Manresa, Universitat de Vic-Universitat Central de Catalunya (Uvic-Ucc), Barcelona, Spain

**Keywords:** Baxter’s nerve, Ultrasound, Heel pain syndrome, Tarsal tunnel

## Abstract

**Purpose:**

Neuropathy of the Baxter nerve (BN) seems to be the first cause of the heel pain syndrome (HPS) of neurological origin.

**Methods:**

41 alcohol–glycerol embalmed feet were dissected. We documented the pattern of the branches of the tibial nerve (TN) and describe all relevant osteofibrous structures. Measurements for the TN branches were related to the Dellon–McKinnon malleolar-calcaneal line also called DM line (DML) for the proximal TT and the Heimkes Triangle for the distal TT. Additionally, we performed an ultrasound-guided injection procedure of the BN and provide an algorithm for clinical usage.

**Results:**

The division of the TN was 16.4 mm proximal to the DML. The BN branches off 20 mm above the DML center or 30 mm distally to it. In most of the cases, the medial calcaneal branch (MCB) originated from the TN proximal to the bifurcation. Possible entrapment spots for the medial and lateral plantar nerve (MPN, LPN), the BN and the MCB are found within a circle of 5 mm radius with a probability of 80%, 83%, and 84%, respectively. In ten out of ten feet, the US-guided injection was precisely allocated around the BN.

**Conclusions:**

Our detailed mapping of the TN branches and their osteofibrous tubes at the TT might be of importance for foot and ankle surgeons during minimally invasive procedures in HPS such as ultrasound-guided ankle and foot decompression surgery (UGAFDS).

## Introduction

Chronic heel pain syndrome (HPS) affects a large number of podiatric patients. The overall incidence of this syndrome is stated between 11% and 15% of the population based on studies of various authors [1, 7, 15, 37, 43, 44]. One overlooked cause for HPS, first described in 1940 by Roegholt [35], might be the entrapment of the first branch of lateral plantar nerve, also known as Baxter’s nerve (BN), the anterior branch of the calcaneal nerve or the inferior calcaneal nerve [3–6, 17, 38]. Thus, the BN might be the most common cause of chronic HPS of neurological origin [3, 5].

Singh et al. described the dorsal extension of the medial border of the plantar fascia (i.e. the deep fascia of the abductor hallucis muscle) as the medial septum (MS) [41]. The topographical localization of this structure is essential for the description of nerve entrapment syndromes at the medial heel region. Heimkes et al. described a connective tissue partition that originates from the medial side of the calcaneus and attaches at the MS forming a middle bridge for the two osteofibrous tubes in which the tibial nerve (TN) branches are running through [16]. Three nerves are running through these osteofibrous tubes, the medial plantar nerve (MPN), the lateral plantar nerve (LPN) and the BN. Singh et al. also stated that this medial intermuscular septum is probably the most important compression site for HPS beside the laciniate ligament (the flexor retinaculum of the ankle) [41].

According to other studies, the entrapment site of the BN, its motor branch for the abductor digiti minimi muscle and its sensitive branch (the latter is also known as the calcaneal branch of the inferior calcaneal nerve) for the periosteum of the medial calcaneal tuberosity can be found in two well-defined sites bounded by osteofibrous structures [2, 24, 38]. The proximal site at the lower calcaneal tube in the distal tarsal tunnel [16], where the nerve runs in between the abductor hallucis fascia and the quadratus plantae muscle [14, 20, 28] and the distal site at the medial calcaneal tuberosity (e.g. plantar fasciopathy, infracalcaneal enthesophyte) [31, 35, 40].

An exact knowledge about this topographic anatomy and the anatomic variability of the branches of the TN in relation to the relevant osteofibrous structures might be a basic requirement for the diagnosis and therapy of the HPS. There is no anatomic study, which verifies all anatomic structures of the TT, which could possibly be involved in nerve entrapments at the TT.

Therefore, the aim of our study was to localize and describe the relationship of these important anatomic structures, which might lead to entrapment neuropathies of the TT and in particular of the lower calcaneal tube, using topographic dissections including ultrasonographic injection procedures’ proofs.

## Materials and methods

### Macroscopic dissection

For our study, we dissected 41 alcohol–glycerol embalmed feet (22 left and 19 rights) from 24 donors (19 male, 22 female). The male donors had an average age of 75 years and an average height of 175 cm. The female donors had an average age of 81 years and an average height of 163 cm.

The individuals had given their written informed consent prior to death for their use for scientific and educational purposes and donated the bodies to the Division of Clinical and Functional Anatomy of the Medical University of Innsbruck [21, 22, 33]. All cadavers were preserved using an arterial injection of an alcohol–glycerol solution and immersion in phenolic acid in water for 1–3 months. The possibility of this solution causing preservation artefacts can be denied [21, 29]. The bodies donated to the Division of Clinical and Functional Anatomy of the Medical University of Innsbruck are a representative sample of the general Austrian population at the age of death [22]. According to Austrian National Law, scientific institutions (in general Institutes, Departments or Divisions of Medical Universities) are entitled to receive the body after death mainly by means of a specific legacy, which is a special form of last will and testament. No bequests are accepted without the donor having registered their legacy and been given appropriate information upon which to make a decision based upon written informed consent (policy of ethics) [22, 26]; therefore, an ethics committee approval is not necessary.

Exclusion criteria of the cadavers were BMI above 30 (impaired ultrasound echogenicity), signs of traumas in the ankle region, a history of ankle or foot ischemic vascular disorders, surgery or space-occupying mass lesions.

**Fig. 1:**
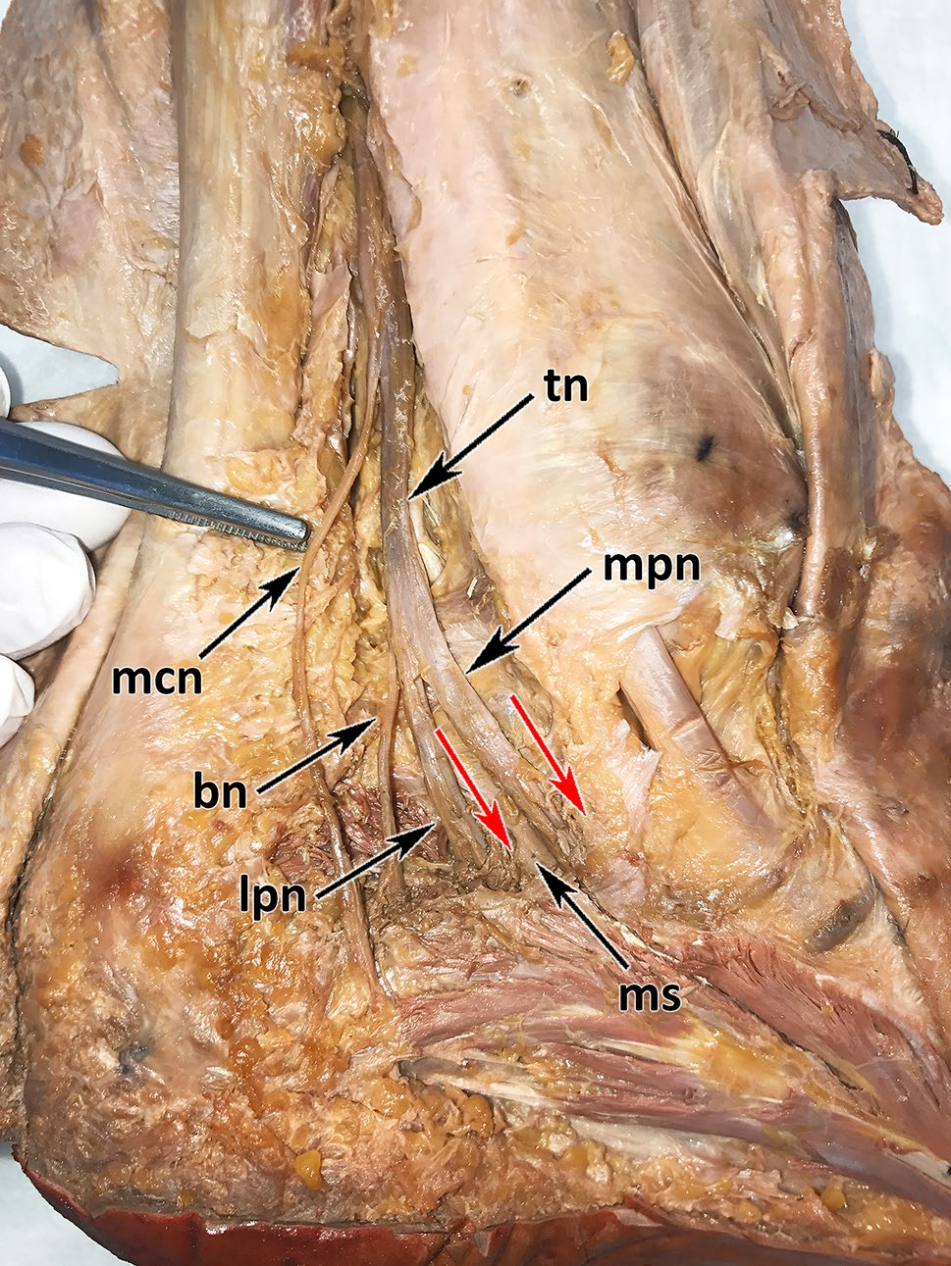
Anatomical dissection of the tibial nerve and its branches running in the TT. *tn* Tibial nerve, *mpn* medial plantar nerve, *lpn* lateral plantar nerve, *bn* Baxter nerve, *mcb* medial calcaneal branch, *ms* medial intermuscular septum, red arrows: nerves entering separated tubes

The medial, retro-malleolar dissection was done 25 cm above the medial malleolus to the proximal third of the plantar compartments (Fig. 1). The skin and soft tissues were removed over the flexor retinaculum and further distal to the sole of the foot. Then the flexor retinaculum, the plantar fascia, the flexor digitorum brevis muscle and the abductor hallucis muscle were cut from their origins. In each specimen, the TN and its terminal branches [MPN, LPN, medial calcaneal branch (MCB) and BN] at the postero-medial TT region were explored and carefully dissected. The medial intermuscular septum was kept untouched to comprehend the exact position, the course and the points of penetration of the nerves to locate possible entrapment spots (Fig. 1). The accompanying vessels were cut off for a better overview and to follow the distal course of the nerves in their osteo-fibrous tubes (Fig. 1). All dissections were made with precise surgical instruments: knife size 4, blade number 20, Stevens’s scissors, and forceps and Metzenbaum scissors. The macroscopic anatomic investigation was made without the use of microsurgical instruments and optical magnification.

**Fig. 2:**
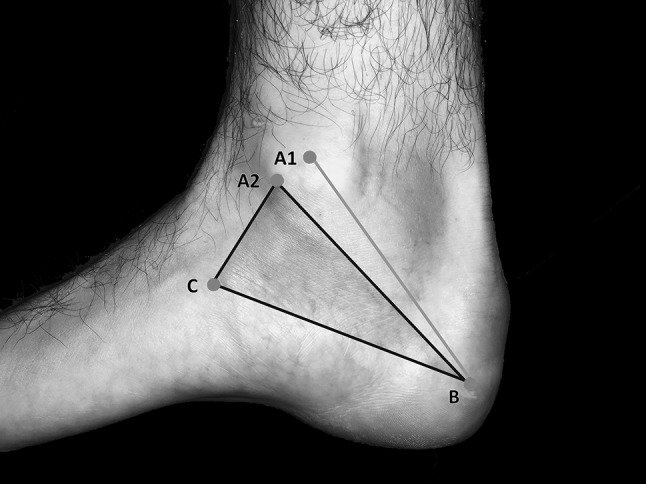
Measurement grid—Dellon–McKinnon malleolar-calcaneal line and Heimkes triangle. Point A_1_: center of the medial malleolus, point A_2_: tip of the medial malleolus, point B: center of the calcaneus, point C: tuberosity of the navicular bone, green line: Dellon–McKinnon malleolar-calcaneal line (DM line, [A_1_B]), black triangle: Heimkes triangle

Our measurements were related to the DML, from the center of the medial malleolus (point A_1_) to the center of the calcaneus (the tip of the calcaneal tubercle at its greatest distance from the medial malleolus), point B and to the Heimkes triangle [11, 16] (Fig. 2). On the malleolar-calcaneal line (A_1_B), we marked the center of the line and measured the distance to the bifurcation of the TN, the branching of the MCB and to the branching of the BN. We examined whether the nerves originated proximal, within or distal to the center of the reference line. Additionally, we measured the distance from the bifurcation of the TN to the origin of the BN. According to the area described by Heimkes [16], we used the isosceles triangle bounded by the following osseous landmarks (Fig. 2): tip of the medial malleolus (point A_2_), the tip of the calcaneal tubercle (point B) and the tuberosity of the navicular bone (point C). We located the MCB, the BN, the LPN and the MPN on the [A_2_B] line. Beginning at the point A_2_ we measured the distances to the nerves crossing the A_2_B line. On the BC line we located the possible entrapment spots affecting the MPN, LPN, BN and the MCB from point C.

All the specimens were positioned at an anatomic position of the ankle (90°) to ensure correct measurements. The measurements were made using a flexible ruler. Documentation was made photographically.

### Ultrasonographic approach and injection procedure

We performed an ultrasound-guided injection of the BN on ten alcohol–glycerol embalmed cadaver feet. We used an ultrasound device with a high-frequency linear probe (8–18 MHz, Sonoscape, Italy).

**Fig. 3:**
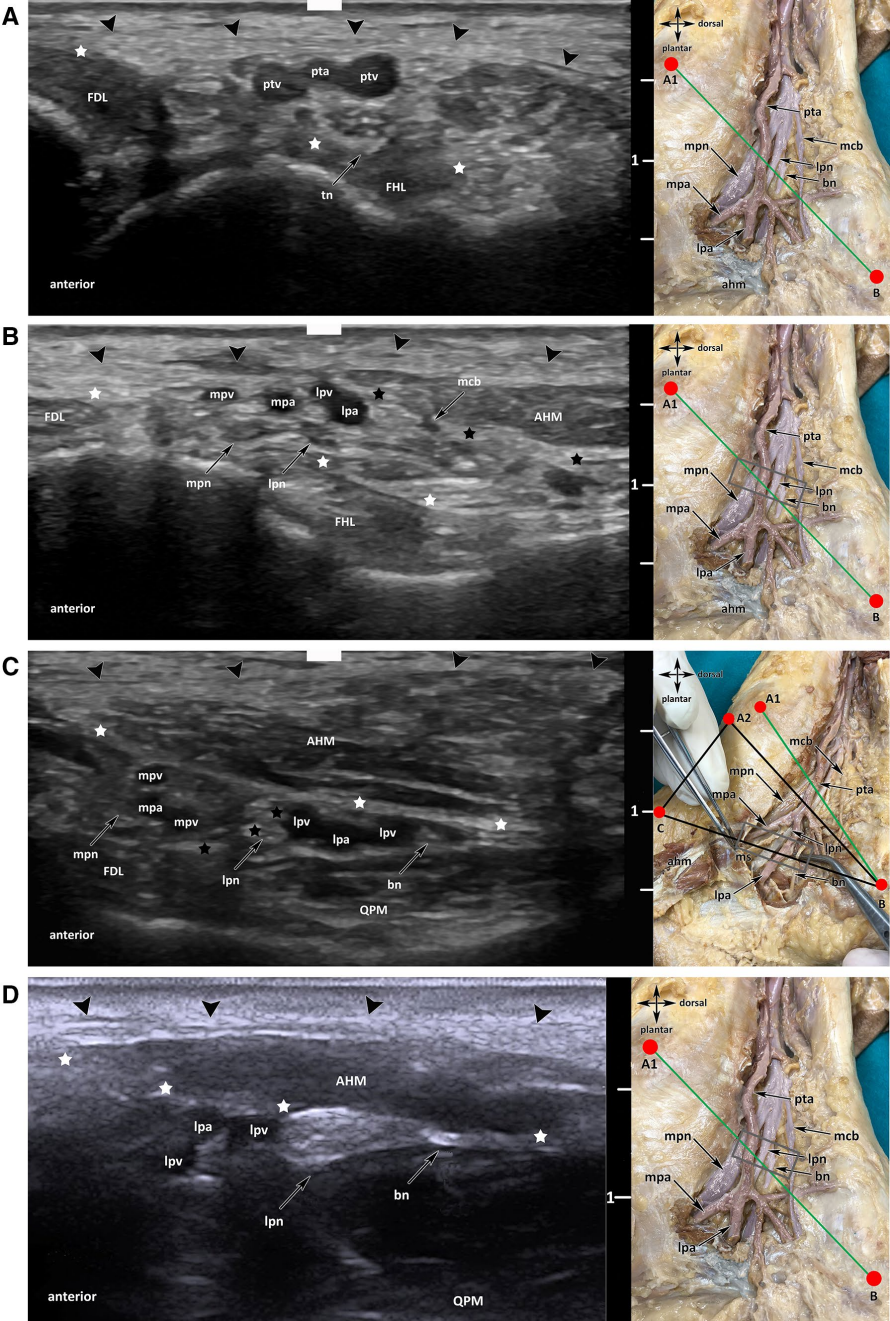
**a** Ultrasound visualization of TN. *tn* tibial nerve, *FDL* flexor digitorum longus, *FHL* flexor hallucis longus, *pta* posterior tibial artery, *ptv* posterior tibial veins, black arrowheads: superficial layer flexor retinaculum, white stars: profound layer flexor retinaculum. **b** Ultrasound visualization of the tibial division and the medial calcaneal branch. *Mpn* medial plantar nerve, *lpn* lateral plantar nerve, *mcb* medial calcaneal branch, *FDL* flexor digitorum longus, *FHL* flexor hallucis longus, *AHM* abductor hallucis muscle, *lpa* lateral plantar artery, *lpv* lateral plantar vein, *mpa* medial plantar artery, *mpv* medial plantar vein, thick arrows:superficial layer flexor retinaculum, white stars:profound layer flexor retinaculum, black stars:medial septum. **c** Ultrasound visualization of the terminal branches (lpn, mpn, bn) on [BC] line. *Lpn* lateral plantar nerve, *mpn* medial plantar nerve, *bn* Baxter’s nerve, *FHL* flexor hallucis longus muscle, *QPM* quadratus plantae muscle, *AHM* abductor hallucis muscle, *mpv* medial plantar vein, *lpv* lateral plantar vein, *mpa* medial plantar artery, *lpa* lateral plantar artery, black arrowheads: superficial layer flexor retinaculum, white stars: medial septum (deep fascia of AHM), black stars: medial septum (interfascicular septum). **d** Ultrasound visualization of the arising of BN on [A_1_B] line. *Lpn* lateral plantar nerve, *bn* Baxter’s nerve, *lpv* lateral plantar vein, *lpa* lateral plantar artery, *AHM* abductor hallucis muscle, *QPM* quadratus plantae muscle, black arrowheads: superficial flexor retinaculum, white star: profound layer flexor retinaculum

**Fig. 4:**
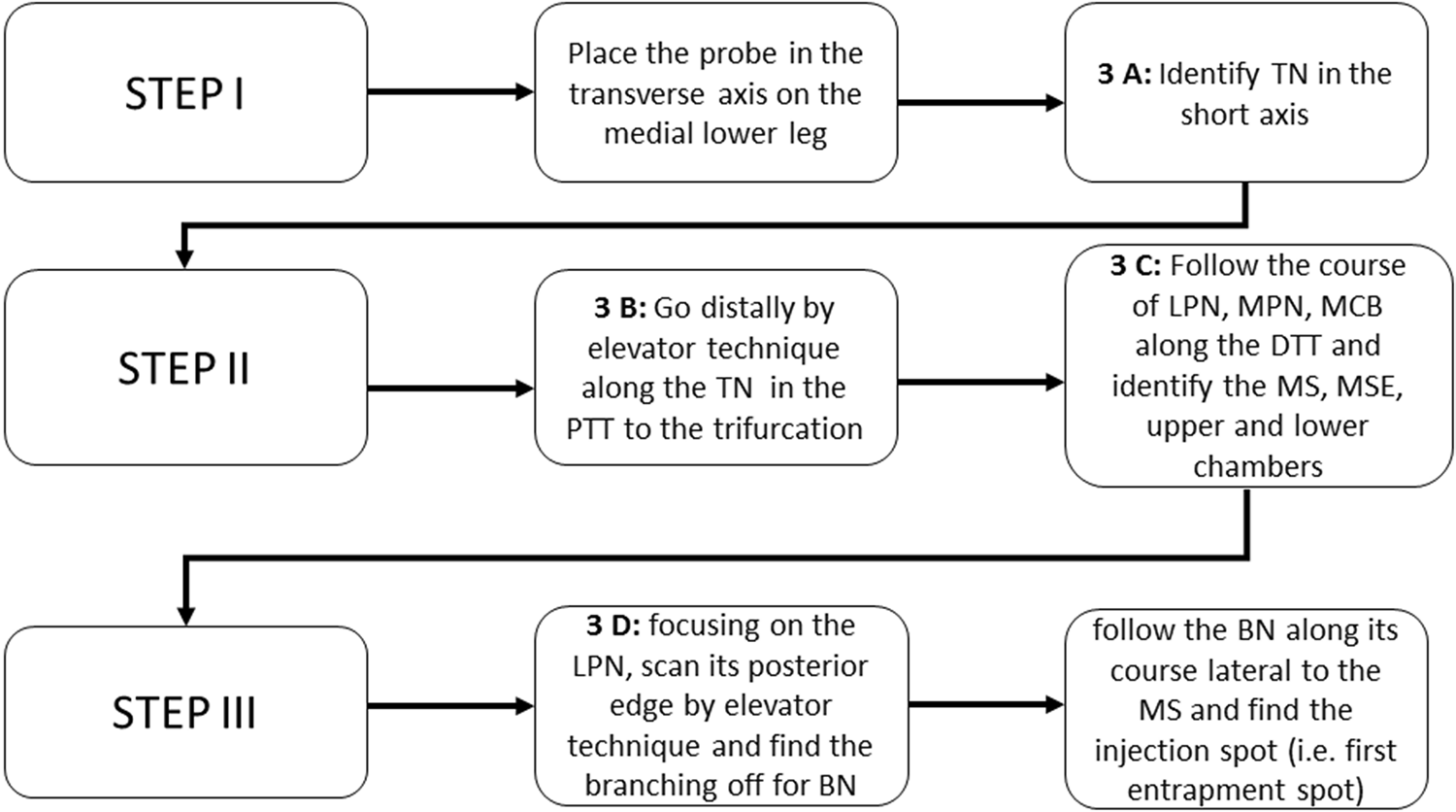
Algorithm for ultrasonography routine implementation. *TN* tibial nerve, *MPN* medial plantar nerve, *LPN* lateral plantar nerve, *BN* Baxter nerve, *MCB* medial calcaneal branch, *MS* medial septum, *MSE* medial septum extension, *PTT* posterior tarsal tunnel, *DTT* distal tarsal tunnel

An ultrasound examination of the region was performed (Fig. 3a–d). To locate all important topographic structures of the medial ankle region, we defined an algorithm for ultrasonographic routine implementation (Fig. 4).

According to Presley et al. [30], the probe was placed on the short axis in a medial retro-malleolar position. The TN has been identified lateral in the depth next to the posterior tibial vascular bundle. Using the elevator technique distal to the trifurcation, each nerve has been examined in the tarsal tunnel. The LPN was found lateral and posterior to the MPN. We followed the nerve on its short axis. Examining its posterior aspect, one should identify the BN as a “monofascicular” nerve of 1–2 mm diameter at this location (hypoechogenic, black “point”; “monofascicular” is meant by an ultrasonographic point of view, not histologically). Following the BN distally it enters its tube (Fig. 3a–d). At this region, the BN typically lies deeper to the medial intermuscular septum (i.e. deep abductor hallucis fascia) and has no accompanying vessels immediately running beside (Fig. 3d).

**Fig. 5:**
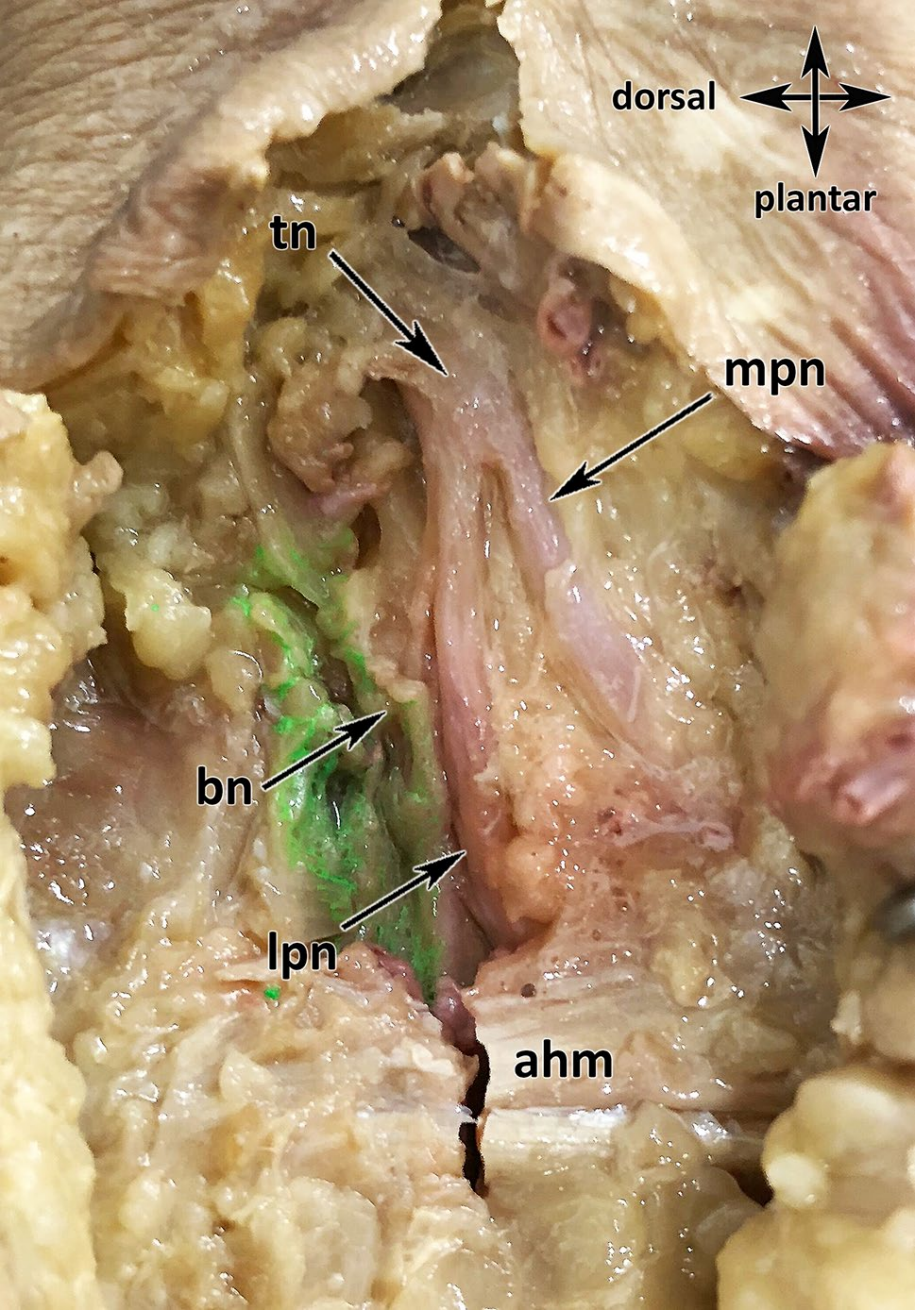
Injection proof: diluted colored latex in BN tube. *tn* tibial nerve, *mpn* medial plantar nerve, *lpn* lateral plantar nerve, *bn* Baxter nerve, *mcb* medial calcaneal branch, *ahm* abductor hallucis muscle

The injection procedure was done using an out-of-plane, posterior-to-anterior approach, slightly oblique in respect to the transverse plane. The ultrasound-guided injection was made with a 27-gauge, 38-mm needle, 0.5-cc syringe and 0.2 cc of 50% diluted colored latex [18]. After the injection, we carefully dissected the layers without spreading the latex to verify if the injective was selectively allocated around the nerve (Fig. 5).

## Results

### Gross anatomical findings

#### Tibial nerve

The divisions of the TN were found at variable levels. The TN divided from 78 mm above the center of the malleolar-calcaneal line to 10 mm distal. On average, in 33 out of 41 specimens, the division of the TN was at 16.4 mm proximal to our reference point (the center of the A_1_B line), in 4 out of 41 within and in 4 out of 41 distal to it. Between the LPN and the MPN we found the extension of the MS, i.e. connective tissue partition of Heimkes et al. in 40 out of 41 feet [16] (Table 1).

**Table 1:** All measurements concerning the reference line [A_1_B]

Specimen	[A_1_B]			Distance^a^
	Tibial division	Branching MCB	Branching BN	
1	40	45	20	20
2	5	170	− 2	7
3	10	15	2.5	7.5
4	0	0	− 5	5
5	7	238	2	5.5
6	27	75	10	17
7	32	35	7	25
8	75	80	10	65
9	− 7	205	− 3	4
		15		
10	8	9	− 7	15
11	78	30	12	66
12	11	120	− 14	25
13	48	30	− 14	62
14	6	55	− 2	8
		55		
15	17	80	2	15
		17		
16	5	25	0	5
17	20	220	10	10
		− 18		
18	10	110	10	0
		35		
19	20	− 15	− 15	35
20	30	4	0	30
21	0	3	− 5	5
22	3	3	− 8	11
23	− 8	170	1.5	9
24	13	85	0	13
25	45	8	− 10	55
26	5	50	− 3	8
27	20	12	0	20
		5		
28	10	30	− 2	12
29	10	40	10	0
30	10	30	10	0
31	40	45	17	23
		45		
32	35	20	/	/
33	13	120	5	8
		35		
34	− 5	20	0	5
		0		
35	− 10	20	− 7	3
36	0	20	2	2
37	0	23	0	0
38	8	20	5	3
		15		
39	5	30	0	5
40	10	35	− 30	40
41	25	5	− 5	30
Average	16.4	49.5	0.1	17.0

All measurements are in mm; negative measurements are distal to reference line; positive measurements are proximal to reference line; measurements = 0 mean lying within the reference line; tibial division = bifurcation of the tibial nerve; *MCB* medial calcaneal branch, *BN* Baxter nerve, *LPN* lateral plantar nerve, *MPN* medial plantar nerve, / no measurement, the nerve was cut off

^a^Distance between the tibial division and the branching off of the BN

#### Medial calcaneal branch

**Fig. 6:**
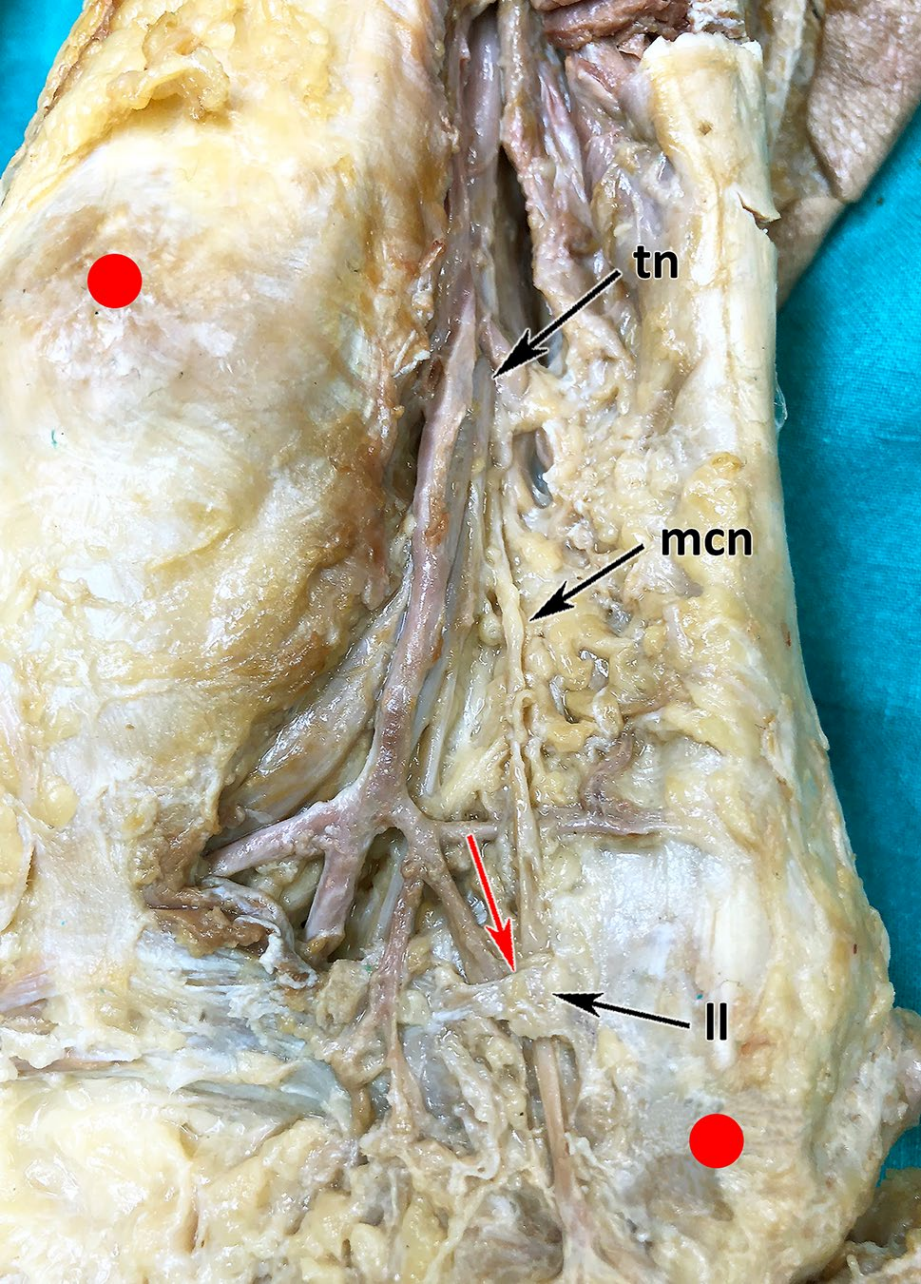
MCB course from depth to surface. *tn* tibial nerve, *mcb* medial calcaneal branch, *ll* extension of lacinate ligament, red arrow: nerve entering tube

The MCB showed a very variable branching pattern. We found the nerve arising from 238 mm proximal to the center of the malleolar-calcaneal line to 18 mm below and calculated a mean distance of 49,5 mm above this reference point. The MCB originated from the TN proximal to the bifurcation in 38 out of 49 cases, at the level of the bifurcation in 3 out of 49 and in 9 out of 49 it branched from the LPN. In 11 out of 41 feed, we found 2 branches of the MCB. The MCB always ran distally to the posteromedial heel in a separate tube from the depth to the surface. This tube is formed by the distal extension of the laciniate ligament (Fig. 6; Table 1).

#### Baxter’s nerve

**Fig. 7:**
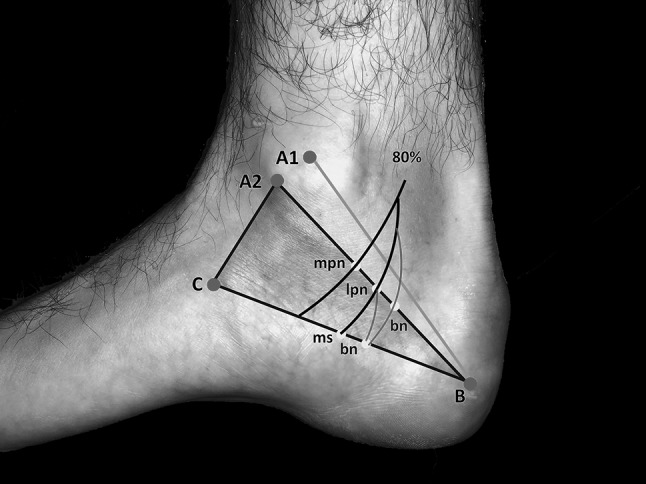
Most common arising of BN (80%). *Mpn* medial plantar nerve, *lpn* lateral plantar nerve, *bn* Baxter nerve, *ms* medial septum, point A_1_: center of the medial malleolus, point A_2_: tip of the medial malleolus, point B: center of the calcaneus, point C: tuberosity of the navicular bone, green line: Dellon–McKinnon malleolar-calcaneal line (DM line, [A_1_B]), black triangle: Heimkes triangle, red branches: most common BN arising out of LPN

The Baxter’s nerve [3–6] showed a more constant branching in comparison to the MCB. It arose 20 mm above the [A_1_B] center to 30 mm distally. In 32 out of 40 specimens, it arose from the LPN (Fig. 7). From the TN, the BN branched off in 4 out of 40 feet proximal to the bifurcation, in 4 out of 40 within the bifurcation. In most cases, the nerve coursed distally in a separate tube after perforating the medial septum (Table 1).

### Topographical spots of the nerves (BN, MCB, LPN, MPN)

On the [A_2_B] line we measured the distance from the tip of the medial malleolus to the MCB, BN, LPN and MPN (crossing points). The average distances are shown in Table 2.

**Table 2:** All measurements concerning the reference line [A2B]

Specimens	[A_2_B]			
	MCB	BN	LPN	MPN
1	55	42	37	30
2	55	50	45	35
3	55	45	40	35
4	60	48	43	38
5	55	45	40	35
6	65	48	45	32
7	65	42	40	33
8	55	52	48	40
9	55	43	40	35
	46			
10	52	47	44	38
11	60	55	50	40
12	55	47	44	40
13	60	50	45	40
14	65	45	42	40
	/			
15	55	47	44	40
	/			
16	60	45	42	35
17	60	40	38	32
	/			
18	/	48	42	35
	60			
19	50	45	40	35
20	70	50	45	38
21	50	45	40	35
22	55	43	40	30
23	50	40	35	30
24	65	50	45	38
25	65	45	40	35
26	70	40	38	32
27	70	60	55	45
	/			
28	70	53	50	40
29	70	53	50	40
30	70	55	50	40
31	55	47	42	35
	/			
32	70	60	55	45
33	70	60	55	45
	/			
34	60	45	42	40
	50			
35	50	42	40	37
36	55	45	40	35
37	55	48	45	40
38	70	55	45	38
	65			
39	55	50	45	40
40	65	50	43	40
41	70	50	43	35
Average		60.0	48.0	43.7

All measurements are in mm; negative measurements are distal to the reference line, positive measurements are proximal to the reference line; measurements = 0 mean lying within the reference line

*MCB* medial calcaneal branch crossing reference line, *BN* Baxter’s nerve crossing the reference line, *LPN* lateral plantar nerve crossing the reference line, *MPN* medial plantar nerve crossing the reference line, / nerve was cut off

**Fig. 8:**
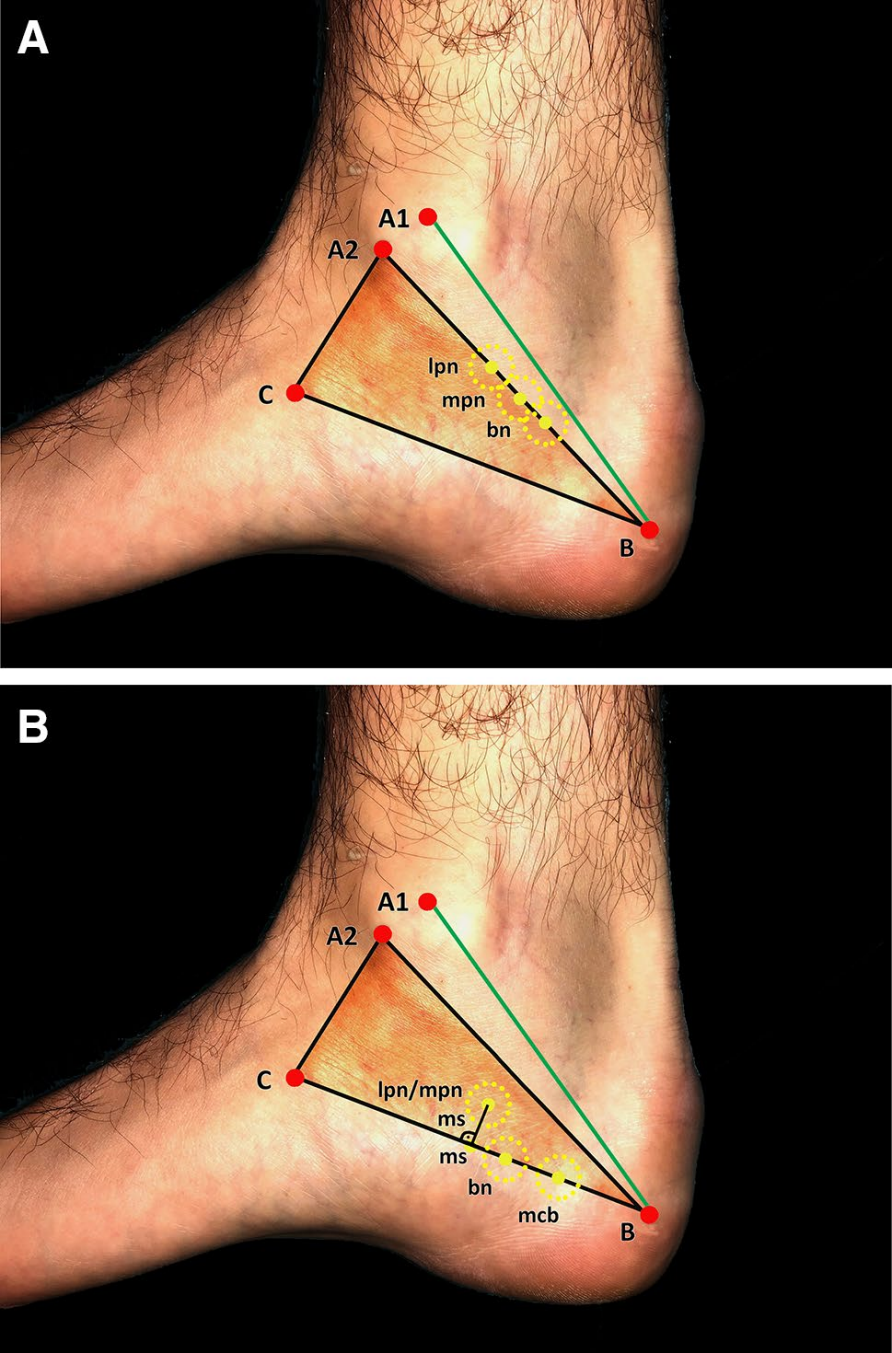
**a** Location spots of LPN, MPN and BN on [A_2_B]. *Mpn* medial plantar nerve, *lpn* lateral plantar nerve, *bn* Baxter nerve, point A_1_: center of the medial malleolus, point A_2_: tip of the medial malleolus, point B: center of the calcaneus, point C: tuberosity of the navicular bone, green line: Dellon–McKinnon malleolar-calcaneal line (DM line, [A_1_B]), black triangle: Heimkes triangle yellow circle: location spot (radius 5 mm). **b** Location spots of LPN/MPN, BN, MCB on [BC]. *Mpn* medial plantar nerve, *lpn* lateral plantar nerve, *bn* Baxter nerve, *mcb* medial calcaneal branch, point A_1_: center of the medial malleolus, point A_2_: tip of the medial malleolus, point B: center of the calcaneus, point C: tuberosity of the navicular bone, green line: Dellon–McKinnon malleolar-calcaneal line (DM line, [A_1_B]), black triangle: Heimkes triangle, yellow circles: location spots (radius 5 mm)

The topographical spots for the BN, LPN and MPN were (Fig. 8a) as follows: mark a point on [A_2_B] at a distance of 50 mm (BN), 45 mm (LPN) or 35 mm (MPN) from the tip of the medial malleolus. Draw a circle (radius 5 mm) around the point of examination. The probability that the searched nerve is within the circle is 73% (BN), 83% (LPN) and 93% (MPN), respectively (Fig. 8a).

The course of MCB was too variable to define a precise point for a regular localization.

### Topographical spots of possible nerve entrapments

**Fig. 9:**
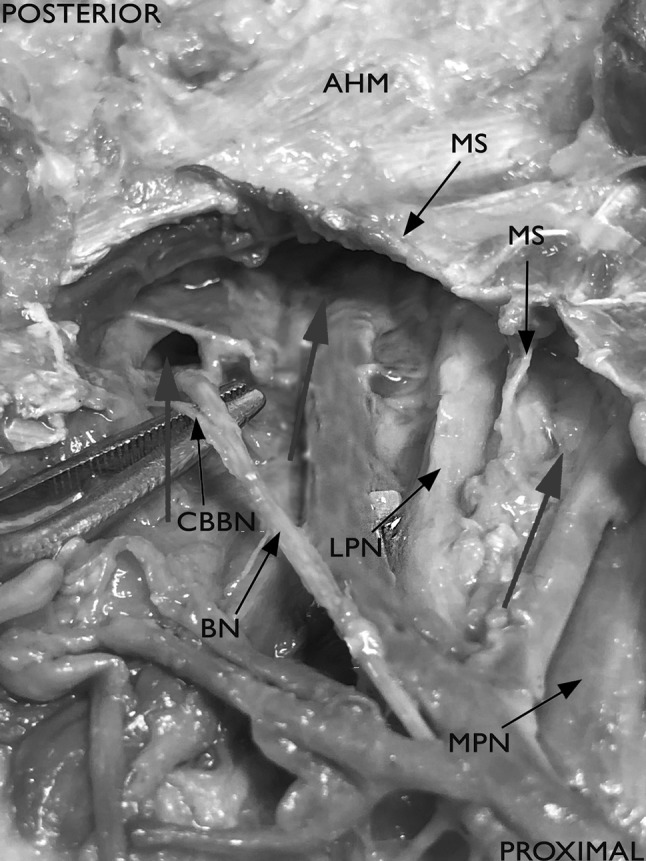
Nerves (LPN, MPN, BN) entering separated tubes, perforating the medial septum. *Mpn* medial plantar nerve, *lpn* lateral plantar nerve, *bn* Baxter’s nerve, *cbbn* calcaneal branch of the Baxter’s nerve, *ms* medial septum, red arrows: nerves entering separated tubes

In most cases, all the examined nerves (MPN, LPN, BN, MCB) coursed in different tubes divided by osteofibrous structures (Fig. 9). For the MCB also the distal extension of the laciniate ligament is a possible entrapment spot (Fig. 6). If the tube of the MCB is too superficial, we can rule out that the medial intermuscular septum is involved.

The topographical spots for a possible entrapment of the MPN, LPN, BN and MCB are as follows (Fig. 8b): mark a point on [BC] line at a distance of 45 mm (LPN/MPN), 50 mm (BN) or 65 mm (MCB) from the tuberosity of the navicular.

If the BN or the MCB is examined, draw a circle (radius 5 mm) around the marked point on [BC] line. If the LPN and the MPN is examined from your marked point (at 45 mm), go 10 mm proximal in a 90° angle to [BC] and mark a second point, draw a circle (radius 5 mm) around the 2nd point (Fig. 8b).

The probability that the possible entrapment spot is within the circle is 80% (LPN/MPN), 83% (BN), 84% (MCB), respectively (Table 3).

**Table 3:** All measurements concerning the reference line [BC] and the possible entrapment spots for the lateral plantar nerve/medial plantar nerve, Baxter’s nerve and the medial calcaneal branch

Specimens	[BC]		Entrapment			
	LPN/MPN	Beginning**	BN	Beginning**	MCB	Beginning***
1	35	20	55	− 17	70	0
2	50	5	50	5	/	/
3	/	/	/	/	/	/
4	43	5	53	0	/	/
5	43	17	60	0	70	3
6	50	8	60	0	80	0
7	42	5	56	0	68	5
8	40	10	47	5	54	0
9	41	10	45	0	58	7
					70	8
10	40	5	/	/	67	− 5
11	42	18	50	6	60	− 5
12	50	5	58	0	72	0
13	50	10	/	/	65	0
14	40	0	45	0	73	− 1
					/	/
15	38	5	52	5	60	0
					/	/
16	38	17	45	7	60	0
17	44	10	/	/	/	/
					/	/
18	40	0	46	0	/	/
					60	0
19	40	8	50	− 10	70	0
20	50	− 8	55	− 3	68	0
21	45	5	/	/	60	0
22	40	5	/	/	60	0
23	45	5	50	3	60	0
24	43	5	50	5	75	− 30
25	40	8	/	/	70	0
26	35	10	/	/	70	0
27	50	15	/	/	70	5
					/	/
28	65	15	/	/	70	6
29	44	8	/	/	70	0
30	50	7	/	/	70	− 3
31	40	10	48	8	55	0
					/	/
32	50	7.5	65	0	70	0
33	45	10	55	0	70	− 5
					/	/
34	40	6	47	0	50	0
					/	/
35	35	6	45	5	70	0
36	40	5	55	0	65	0
37	40	0	50	0	55	− 6
38	40	12	55	0	60	0
					/	/
39	45	10	50	0	63	0
40	38	10	45	0	70	0
41	40	10	45	0	65	7
Average	43.2	8.0	51.3	0.7	65.6	− 0.4

All measurements are in mm; negative measurements are distal to reference; positive measurements are proximal to the reference line; measurements = 0 mean lying within the reference line

*LPN/MPN* lateral plantar nerve and medial plantar nerve; *BN* Baxter’s nerve, *MPN* medial plantar nerve, *MCB* medial calcaneal branch

**Beginning of the medial intermuscular septum directly next to the nerves (LPN/MPN or BN)

***Beginning of the laciniate ligament directly next to the MCB

The frequency of occurrence, the average localization on the [BC] line and the proximal beginning of relevant septal structures affecting the MPN or LPN, BN or MCB are shown in Table 3.

### Ultrasonographic-guided injection procedure of the Baxter’s nerve

We were able to find all nerves (MPN, LPN, BN, MCB) within the circular location spots on [A_2_B] and also able to find the nerve penetration spots through the medial intermuscular septum within the circle on the [BC] line (Fig. 8a, b). The parts of the medial intermuscular septum were ultrasonographically not visualizable, except the part of the medial intermuscular septum that divides the upper and lower tube (Fig. 9). After dissection of the BN, previously infiltrated, we have ascertained that in ten out of ten feet the injectate was selectively allocated around the nerve (Fig. 5).

## Discussion

The heel pain syndrome (HPS) is a frequent condition among the MSK and in particular podiatric pathologies [1, 7, 15, 43, 44]. The neuropathy of the Baxter nerve is described with prevalence between 15–20% in the literature, is very relevant among the plantar HPS in general and has been described to be the first cause of plantar HPS with neurological origins [3, 5, 28, 34]. It can appear as an isolated condition or in combination with other kind of heel pain, such as plantar fasciopathy, depending on the location of the entrapment [1]. The Tinel sign elicits pain due to nervous sprouting resulting from chronic compression and consequent axonal demyelination [1]. Although this sign may give rise to a false-negative result, in early or late conditions of nerve compression, it remains the most common sign in clinical diagnosis of tarsal tunnel syndromes [1]. In our study, we obtained topographical points for the localization of the TN branches in the TT, with high accuracy, between about 74% and 93% in a cluster of 41 feet. Those points could be crucial in possibly increasing the diagnostic accuracy of clinical tests such as the Tinel sign for tarsal tunnel syndrome.

Fealey et al. demonstrated that half of the asymptomatic, non-diabetic average population, older than 45 years, have abnormal electrodiagnosis in the distal tarsal tunnel [13]. Recht et al. stated that the prevalence of an atrophy of the abductor hallucis muscle is supposed to be an indirect diagnostic proof for Baxter’s neuropathy in MRI; this has been observed in 6% of the general asymptomatic population [32].

Fortunately, since 1991, the diagnostic ultrasound has been established as a validated procedure for the diagnosis of peripheral nerve entrapments such as for the carpal tunnel syndrome [10].

More recently, it has been demonstrated that high-resolution ultrasonography (more than 12 MHz) seems to be a promising tool for the diagnosis of nerve entrapments and could support the treatment of interventional procedures in complex anatomical ankle and foot regions such as the tarsal tubes [19, 25, 30].

In our study, we described a detailed topographical neuroanatomic mapping of the TN division at the proximal and distal tarsal tunnels in relation to the most common osteofibrous structures involved in entrapment to make the diagnosis and treatment of this complicated syndrome more accurate.

Heimkes et al. dissected 60 feet and found a connective tissue structure in 55 specimens, attached on the deep fascia of the abductor hallucis muscle, originating from the calcaneus or from the flexor hallucis longus sheet, between the lateral and medial plantar nerves [16]. We assessed this connective tissue as a part of the medial intermuscular septum which we found in almost every foot.

Ling et al. defined in 2008 three vertical fascial septa in the sole of the foot [23]: the lateral septum, the intermediate septum and the medial septum. We focused our examination on the medial intermuscular septum (the dorsal extension of the medial border of the plantar aponeurosis), because of its convenient location inside the Heimkes triangle and, furthermore, its great clinical relevance: LPN, MPN, and the BN run deep and next to this intermuscular septum. The medial intermuscular septum might be a possible point of compression for the nerves running through the tarsal tubes (LPN, MPN, and BN), if pathological anomalies occur.

Brown and Singh et al. reported this common point of entrapment for the BN [9, 41]: between the medial intermuscular septum (the deep fascia of the abductor hallucis muscle) and the quadratus plantae muscle, more precisely at the hiatus, where the nerve enters its osteofibrous tube (Fig. 8).

Singh and Kumar et al. dissected 19 feet and documented the distance of the nerve points of penetration through the medial intermuscular septum from the posterior border of the calcaneus (average distances of LPN 4.1 cm ± 1.8 cm; MPN 5.6 cm ± 1.6 cm). We agree and our results also confirm these distances of the terminal branches of the TN [41].

Despite the limited number of feet in this study [41], we can conclude that the clinically relevant structures follow a clear regularity, which makes the application of our “circle method” possible for clinical use for the diagnosis and treatment of tarsal tunnel syndromes.

As already described by Heimkes et al., we also observed that almost always the vessels run superficially to the nerves inside the osteofibrous tubes [16]. The space in these tubes is very limited, thus the danger of a nerve entrapment is very high as blood vessels and the osteofibrous structures could compress the nerves (Fig. 3a–d).

Our ultrasound-guided injection procedure, which allocated the selectively around the BN, and our ultrasonographic results, which could identify the BN in the lower calcaneal tube with great accuracy, confirm that high-resolution ultrasound guidance for minimally invasive procedures could be possible, accurate and at the same time safe. Presley et al. in their study could show similar results except for the overflow of the injectate [30]. To surround the nerve selectively, as described by Thallaj et al., we used less than 0.3 ml of injective; this quantity usually is used in clinical practice for little peripheral nerves, which lie within proper fascial compartments [42].

Once precise diagnosis has been made through ultrasonography, and conservative therapy has been failed, the results of our study might support the hypothesis that podiatrists and foot and ankle surgeons, trained in ultrasonography, could use ultrasound-guided, minimally invasive ankle and foot decompression surgery procedures (UGAFDS) for a secure, safe and accurate tarsal tunnel release. This must be proven in further studies, which are already under construction.

Nevertheless, we urge, before using more invasive procedures, that one also tries less invasive therapeutic procedures such as selective ultrasound-guided hydro-dissection for each calcaneal tube for tarsal tunnel syndromes. This technique consists of high-volume injection of fluid under ultrasound guidance to “dissect” the anatomic planes and tissue spaces to decompress nerves [8]. Fader et al. and Bokey et al. reported the proven safety, speed of response and the low costs of this treatment [8].

The study of Sammarco et al. showed good outcomes (between 71 and 91%) after open decompression surgery of the proximal tarsal tunnel; however, one has to keep in mind that when a space-occupying mass was not present, the percentage rate of success of this procedure has been estimated to be less encouraging [39].

Franson et al. also pointed out that one might achieve better results after modifying the technique including a more extensive and distal release of the TN and its branches at the calcaneal tubes of the distal tarsal tunnel [12]. Minimally invasive surgery for tarsal tunnel syndrome revealed the same good outcomes as open surgery as presented by El Shazly et al., at the same time minimizing soft-tissue dissection, potential wound complications and scar fibrosis reducing offloading and patient recovery time [12].

In 2011, McShane et al. described, for the first time, an ultrasound-guided procedure for carpal tunnel release with remarkable outcomes [27].

This type of ultrasound-guided minimally invasive procedures for TT release, because of its ultra-minimally invasive nature, might reduce the typical surgical postoperative disadvantages of an open release, has less complications and, as reported by Mc Shane et al., return to work activities for patients up to four times faster even compared to endoscopic procedures [27].

In the light of the abovementioned results collected for minimally invasive surgeries of the TT, like the ultra-minimally carpal-tunnel release technique described by McShane et al. [36]. We hope that minimally invasive ultrasound ankle and foot decompressions surgery techniques (UGAFDS) for the proximal and distal TT for patients with heel pain syndrome will be described, based on the anatomical results of this study.

Our detailed findings of the TN divisions, osteofibrous tubes and septa at the tarsal tunnel with ultrasound-guided BN injection proof expand the possibilities of minimally invasive procedures for selective diagnostic tibial branches nerve blocks at the TT.

## Conclusion

Our detailed topographical mapping of the TN branches and their osteofibrous tubes at the proximal and distal tarsal tunnel might be of importance for ankle and foot practitioners and especially for surgeons during minimally invasive procedures in heel pain syndrome, such as ultrasound-guided ankle and foot decompression surgery (UGAFDS).
